# Diverticulite duodénale: complication inhabituelle pas toujours facile à gérer

**DOI:** 10.11604/pamj.2015.22.259.7784

**Published:** 2015-11-19

**Authors:** Abderrahman Elhjouji, Lamine Jaiteh, Ahmed Bounaim, Abdelmounaim Aitali, Khalid Sair

**Affiliations:** 1 Service de Chirurgie Viscérale I, Hôpital Militaire d'Instruction Mohamed V, Rabat, Maroc

**Keywords:** Diverticule, duodénum, infection, diverticulum, duodenum, infection

## Abstract

Les diverticules duodénaux sont assez fréquents, la majorité reste asymptomatique. Les complications les plus fréquemment rapportées sont les hémorragies et les pancréatites. Contrairement aux diverticules coliques, la survenue de diverticulite est rare. Nous rapportons le cas d'une infection d'un gros diverticule duodénal en mettant le point sur la difficulté de la prise en charge de cette entité pathologique.

## Introduction

L'incidence des diverticules duodénaux (DD) est mal connue. Elle varie entre 2 et 23% dans les séries d'autopsie et d'endoscopie [1]. Dans la majorité de cas ils sont asymptomatiques [2]. A travers cette observation, nous rapportons le cas d'une diverticulite duodénale et nous mettons le point sur les difficultés de prise en charge de cette complication inhabituelle.

## Patient et observation

**Figure 1:**
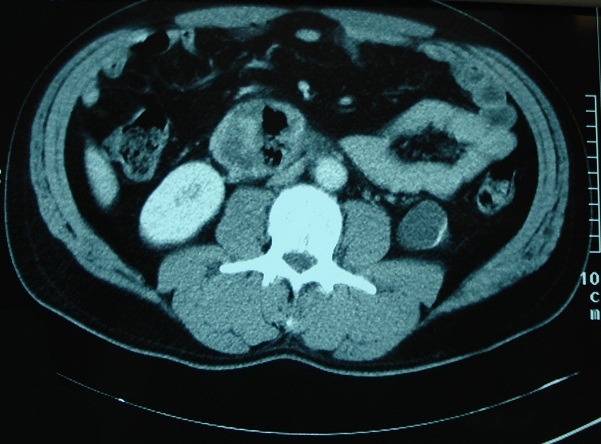
Coupe scannographique montrant le diverticule à contenu hétérogène avec de l'aire à l'intérieur

**Figure 2:**
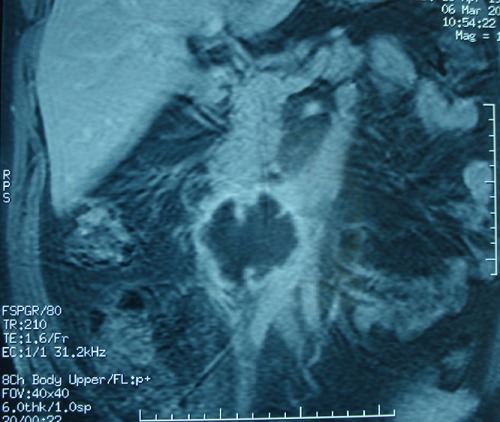
Coupe coronale d'IRM montrant le diverticule duodénal en hyposignal T1

**Figure 3:**
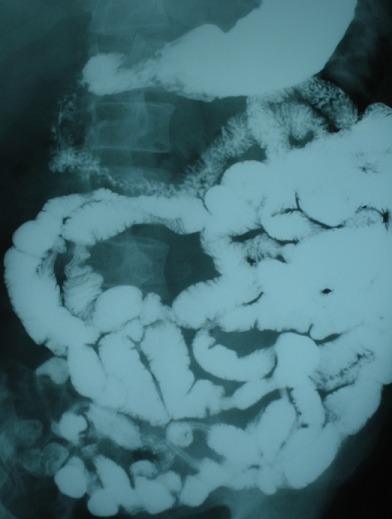
TG montrant le refoulement des anses proximales par le diverticule sans extravasation de produit de contraste

**Figure 4:**
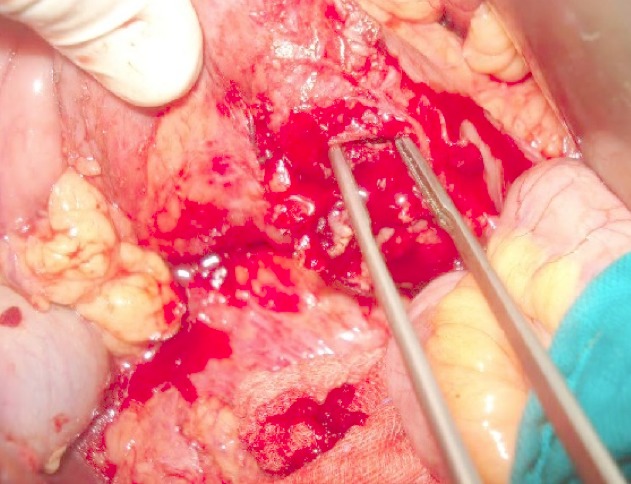
Image opératoire montrant le moignon diverticulaire après sa résection

Patient G.M, âgé de 55 ans, sans antécédent pathologique notable. Admis aux urgences pour douleurs abdominales au niveau de l'hypochondre, et du flanc droit, associées à une fièvre à 38,5°C. L'examen clinique trouvait une légère défense abdominale épigastrique. Le taux des globules blancs était de 18000/mm^3^. Le scanner abdominal a mis en évidence une masse hypodense, hétérogène, de contours irréguliers, à paroi épaissie, avec une infiltration inflammatoire et de l'aire à l'intérieur, mesurant 5 cm de diamètre du grand axe (Figure 1). Cette masse semblait être au dépend du 3ème duodénum. Il n'y avait pas de pneumo ni de retropneumopéritoine ni d’épanchement intra abdominal. L'IRM a confirmé les données du scanner en montrant une structure hétérogène de contours irrégulier, hyposignal en T1 (Figure 2), et hypersignal en T2. Les structures vasculaires étaient épargnées, l'uretère droit passait juste à côté sans qu'il soit pris par la masse. La fibroscopie oesogastroduodénale (FOGD) n'a pas montré de lésion à part une muqueuse duodénale légèrement érythémateuse. Le transit du grêle (TG) a objectivé un aspect d’écartement des anses intestinales par le diverticule sans signe d'extravasation extradigestive (Figure 3). En absence d'une réponse favorable au traitement médical associant l'antibiothérapie (Amoxicilline, acide clavulanique, amikacine, métronidazole) et l'alimentation parentérale, le patient fut opéré, l'exploration chirurgicale a objectivé la présence d'un diverticule abcédé, se développait sur le bord externe du genu inferius. La mobilisation du bloc duodénopancréatique était délicate à cause du processus inflammatoire, la dissection du diverticule était menée de façon très prudente après repérage des structures vasculaires. On a opté pour une résection diverticulaire avec suture en 2 plans du pertuis duodénal (Figure 4). Les suites étaient marquées par une fistule digestive à gros débit qui a nécessité une reprise chirurgicale à j + 5. Une exclusion duodénale a été réalisée. L’évolution était favorable. L'examen de contrôle après un mois était sans particularité.

## Discussion

Le duodénum est la 2^ème^ localisation des diverticules intestinaux après le côlon [3]. Son incidence exacte est mal connue, elle varie de 2 à 5% dans la population générale [1]. Dans 90% des cas, les diverticules duodénaux sont asymptomatiques [2]. Les complications sont rares mais graves (Perforation, hémorragie, obstruction biliaire ou duodénale, pancréatite, diverticulite) [3]. Dans l’étude de Akhrass qui a inclus 208 cas de diverticule duodénal et de l'intestin grêle, il y a eu une prédominance de complications hémorragiques pour les diverticules duodénaux, alors que la perforation était plus fréquente en cas de diverticule de l'intestin grêle [4]. Le caractère non spécifique de ces complications est souvent responsable d'un retard diagnostique, et par conséquence un retard de prise en charge thérapeutique qui peut engager le pronostique vital des patients [5]. Notre observation illustre les difficultés qu'on peut rencontrer pour diagnostiquer une complication exceptionnelle du DD qui est la diverticulite. La radiologie était d'un apport considérable, elle nous a permis grâce aux images de reconstruction scannographique (Figure 1) et aux coupes coronales de l'IRM (Figure 3, 4) de mettre en évidence le diverticule duodénal. Au fait, un diverticule avec un petit collet risque de passer inaperçu à l'endoscopie. L'inflammation entraînerait un épaississement muqueux avec obstruction de la communication avec la lumière duodénale. L'opacification digestive peut ne pas montrer l'extravasation du produit de contraste, par contre elle peut mettre en évidence des images de refoulement des anses intestinales [6] surtout pour les diverticules de grande taille. Son utilisation est limitée dans le contexte de l'urgence, si une cure chirurgicale est prévue [5]. En absence du traitement l’évolution peut se faire vers la perforation ou l'hémorragie [6]. Le scanner permet un diagnostique précoce de la lésion, ce qui permet de proposer un traitement adapté au stade de la maladie et au terrain. On peut proposer un traitement médical fait d'antibiothérapie et d'alimentation parentérale, à condition que la surveillance soit rapprochée en milieu chirurgical [3, 7]. En cas d’échec de cette option, un traitement chirurgical radical doit être entrepris dans les plus brefs délais. Il fait appel à une diverticulectomie, avec fermeture duodénale de préférence en 2 plans, nécessitant parfois une suture de tissu inflammatoire, ce qui expose au risque de fistule ou de péritonite post-opératoire [1, 3].

## Conclusion

La diverticulite duodénale est une complication rare, pas toujours anodine. Elle pose beaucoup de problèmes diagnostiques et de prise en charge thérapeutique. Les examens radiologiques et notamment la TDM abdominale permettraient un diagnostic à un stade utile pour proposer un traitement adéquat.
